# Exploring the Relationship between Gut Microbiome Composition and Blood Indole-3-acetic Acid in Hemodialysis Patients

**DOI:** 10.3390/biomedicines12010148

**Published:** 2024-01-10

**Authors:** Ping-Hsun Wu, Yu-Fang Tseng, Wangta Liu, Yun-Shiuan Chuang, Chi-Jung Tai, Chun-Wei Tung, Kean-Yee Lai, Mei-Chuan Kuo, Yi-Wen Chiu, Shang-Jyh Hwang, Wei-Chun Hung, Yi-Ting Lin

**Affiliations:** 1 Division of Nephrology, Department of Internal Medicine, Kaohsiung Medical University Hospital, Kaohsiung Medical University, Kaohsiung 807, Taiwan; 970392@kmuh.org.tw (P.-H.W.); mechku@kmu.edu.tw (M.-C.K.); chiuyiwen@kmu.edu.tw (Y.-W.C.); sjhwang@kmu.edu.tw (S.-J.H.); 2 Faculty of Medicine, College of Medicine, Kaohsiung Medical University, Kaohsiung 807, Taiwan; 3 Center for Big Data Research, Kaohsiung Medical University, Kaohsiung 807, Taiwan; kinkipag@gmail.com; 4 Research Center for Precision Environmental Medicine, Kaohsiung Medical University, Kaohsiung 807, Taiwan; 5 Department of Family Medicine, Kaohsiung Municipal Hsiao-Kang Hospital, Kaohsiung Medical University Hospital, Kaohsiung Medical University, Kaohsiung 807, Taiwan; jackenmina@gmail.com; 6 Department of Biotechnology, Kaohsiung Medical University, Kaohsiung 807, Taiwan; liuwangta@kmu.edu.tw; 7 Department of Family Medicine, Kaohsiung Medical University Hospital, Kaohsiung 807, Taiwan; taichijung@gmail.com; 8 Institute of Biotechnology and Pharmaceutical Research, National Health Research Institutes, Miaoli 350, Taiwan; cwtung@nhri.edu.tw; 9 Graduate Institute of Data Science, College of Management, Taipei Medical University, Taipei 110, Taiwan; 10 Post Baccalaureate Medicine, Kaohsiung Medical University, Kaohsiung 807, Taiwan; laikeanyee@gmail.com; 11 Department of Microbiology and Immunology, College of Medicine, Kaohsiung Medical University, Kaohsiung 807, Taiwan

**Keywords:** indole-3-acetic acid, microbiome, hemodialysis, end-stage kidney disease

## Abstract

Indole-3-acetic acid (IAA), a protein-bound uremic toxin resulting from gut microbiota-driven tryptophan metabolism, increases in hemodialysis (HD) patients. IAA may induce endothelial dysfunction, inflammation, and oxidative stress, elevating cardiovascular and cognitive risk in HD patients. However, research on the microbiome–IAA association is limited. This study aimed to explore the gut microbiome’s relationship with plasma IAA levels in 72 chronic HD patients aged over 18 (August 2016–January 2017). IAA levels were measured using tandem mass spectrometry, and gut microbiome analysis utilized 16s rRNA next-generation sequencing. Linear discriminative analysis effect size and random forest analysis distinguished microbial species linked to IAA levels. Patients with higher IAA levels had reduced microbial diversity. Six microbial species significantly associated with IAA levels were identified; *Bacteroides clarus*, *Bacteroides coprocola*, *Bacteroides massiliensi*, and *Alisteps shahii* were enriched in low-IAA individuals, while *Bacteroides thetaiotaomicron* and *Fusobacterium varium* were enriched in high-IAA individuals. This study sheds light on specific gut microbiota species influencing IAA levels, enhancing our understanding of the intricate interactions between the gut microbiota and IAA metabolism.

## 1. Introduction

Indole-3-acetic acid (IAA) belongs to the family of indolic uremic solutes and is a protein-bound uremic solute resulting from tryptophan metabolism [1]. Its levels have been observed to increase in patients with chronic kidney disease (CKD), with higher serum concentrations found in individuals with stage 3–5 CKD and undergoing hemodialysis compared to healthy controls [1]. Recent studies have implicated IAA in inducing endothelial dysfunction, inflammation, and oxidative stress, thereby raising cardiovascular risk in CKD patients [1,2]. However, IAA has shown promise as a potential therapeutic agent in certain contexts as it mitigates hepatotoxicity in mice by reducing hepatic lipogenesis, as well as ameliorating oxidative and inflammatory stress [3]. Notably, IAA has been linked to anxiety and depressive symptoms in CKD patients [2] and cognitive function decline in hemodialysis patients [4], highlighting the complex interplay between this compound and systemic health. Additionally, IAA induced an elevation in tissue factor expression in human endothelial cells, peripheral blood mononuclear cells, and vascular smooth muscle cells through the activation of the aryl hydrocarbon receptor (AHR) [5].

The human gut microbiome is a complex community of microorganisms that live in the gastrointestinal system [1]. There have been observed changes in the gut microbiome in patients with CKD and those undergoing hemodialysis [6,7,8,9,10,11]. CKD patients tend to have decreased microbiota diversity compared to healthy individuals, suggesting an altered gut microbial ecosystem [6]. Studies have also investigated the gut microbiota composition in patients undergoing hemodialysis or peritoneal dialysis [6,7,8], showing that gut dysbiosis or an imbalance in the gut microbiota caused by dialysis is associated with inflammation and adverse outcomes in patients with CKD [6,7,9]. However, more research is needed to fully understand the complex relationship between the gut microbiome and CKD or hemodialysis patients. Larger clinical studies are required to establish more comprehensive patterns and implications [6,7,9].

IAA, as a microbial tryptophan metabolite, plays a crucial role in influencing the intestinal microbiota and has the potential to impact various systems in patients with CKD [12,13,14]. Several *Bacteroides* species, including *Bacteroides thetaiotaomicron*, *Bacteroides eggerthii*, and *Bacteroides ovatus*, as well as *Clostridium* sp., such as *Clostridium difficile* and *Clostridium perfringens*, have been documented to produce IAA in healthy populations [15,16,17]. Despite its significance, there remains a notable lack of research concerning the specific association between IAA and the gut microbiome in CKD patients. Addressing this research gap, the current study sought to explore the connections between the gut microbiome and blood IAA in hemodialysis patients, shedding light on the intricate interplay between IAA, the gut microbiome, and kidney disease.

## 2. Materials and Methods

### 2.1. Study Participants

This research was carried out at the dialysis units of Kaohsiung Medical University and Kaohsiung Municipal Hsiao-Kang Hospital spanning from August 2016 to January 2017. A total of 72 participants, aged over 18 years and undergoing hemodialysis for more than 90 days, were defined as long-term dialysis and enrolled. All participants underwent regular hemodialysis using high-efficiency dialyzers three times weekly, with blood and dialysate flow rates set at 250–300 mL/min and 500 mL/min, respectively, during each 3.5 to 4 h session. The study protocol (KMU-HIRB-E(I)-20160095 and KMUHIRB-E(I)-20190189) was approved by the Institutional Review Board of Kaohsiung Medical University, and written informed consent was obtained from all participants.

### 2.2. Comorbidity, Laboratory, and Clinical Variables

Patient data, encompassing anthropometric details, gender, age, medical history, and biochemical information, were extracted from the electronic healthcare system within each hemodialysis unit. Blood samples were collected from the arteriovenous fistula or graft before the scheduled hemodialysis session, following an overnight fasting period. Biochemical analyses comprised assessments of albumin, low-density lipoprotein (LDL) cholesterol, fasting glucose, blood urea nitrogen (BUN), creatinine, uric acid, sodium, potassium, total calcium, phosphate, and normalized protein catabolic rate (nPCR). Additionally, fecal and blood samples were collected.

### 2.3. IAA Measurement by Tandem Mass Spectrometry Laboratory

IAA levels were evaluated utilizing an Agilent 1200 High-Performance Liquid Chromatography (HPLC) system (Agilent Technologies, Palo Alto, CA, USA) connected to an API 4000Q triple-quadrupole mass spectrometer (API 4000QTrap, Applied Biosystems/MDS SCIEX, Concord, ON, Canada) with an electrospray ionization (ESI) source in positive ion mode. A Kinetex Phenomenex C8 column (250 mm × 4.6 mm × 5 m) was used for chromatographic separation at room temperature using a mobile phase of 5% acetonitrile with 0.1% formic acid (solvent A) and 95% acetonitrile with 0.1% formic acid (solvent B). Standard IAA (Sigma-Aldrich, Catalogue #15148, St. Louis, MO, USA) solutions were prepared in acetonitrile and diluted to 10,000 g/mL as stock solutions. For each analytical batch, calibration standards were created using a confined mixture of serial concentrations ranging from 0.012 to 25 g/mL in IAA. These standards were refrigerated at 4 °C throughout the study, while human serum samples were stored at −20 °C.

The samples were prepared by precipitating the proteins with acetonitrile for 8 min, then centrifuging them at 13,400× *g* and 4 °C before transferring the supernatants to clean 1.5 mL Eppendorf tubes. The sample was diluted ten times in Solvent A, vortexed for ten seconds, and then filtered through a 0.22 m PVDF filter into the injection vial before analysis on the LC–MS/MS apparatus. Typical fragmentation transitions were employed together with the multiple reaction monitoring (MRM) mode for measurements of the IAA standard, the MRM transitions, the de-clustering potential, the collision energy, and the collision cell exit potential. Applied Biosystems Analyst (1.4.2) software was used for data collection and quantitative analysis.

### 2.4. Bacterial 16S rRNA Amplicon Sequencing

All fecal samples were promptly frozen upon collection and transported in cooler bags to the laboratory (Germark Biotechnology, Taichung, Taiwan) within 24 h. DNA extraction was performed using the QIAamp DNA Stool Mini Kit (Qiagen, Germantown, MD, USA), and an amplicon library was generated by amplifying the variable regions 3 and 4 (V3–V4) of the 16S rRNA gene with barcode-indexed PCR primers (341F and 805R) [18]. To prevent batch effects, the amplicons were sequenced (300 bp paired-end) using an Illumina MiSeq sequencer (Germark Biotechnology, Taichung, Taiwan). The data were preprocessed before importing the raw sequence data into Quantitative Insights Into Microbial Ecology 2 (QIIME2) and involving adapter removal to ensure the cleanliness of the data. Then, the paired-end reads were merged and denoised using Divisive Amplicon Denoising Algorithm 2 (DADA2), which yielded precise amplicon sequence variants (ASVs). Taxonomy classification of ASVs was conducted utilizing the SciKit Learn-based approach [19], employing a search within the SILVA reference database (release v138, trimming to V3–V4 region) [20]. The bases were filtered out with a Phred quality score below 30 to preserve data quality.

### 2.5. Statistical Analysis

Percentages or mean ± SD were used to present the demographic characteristics. A chi-square test or independent *t*-test was used to compare the differences between categorical or continuous variables of individuals with different IAA levels, respectively. Patients were divided into two groups based on the median value of IAA, which was 0.5 µg/mg. The linear discriminant analysis of effect size (LEfSe) analysis [21] and the random forest method [22] were applied to explore the bacterial community differences between individuals with varying levels of IAA. The logarithmic linear discriminant analysis (LDA) scores for discriminative IAA groups and the significance level for the non-parametric factorial Kruskal–Wallis test were set to 2 and 0.05, respectively. All statistical analyses were conducted using R statistical software (version 3.5.1) with a *p*-value less than 0.05 considered statistically significant.

## 3. Results

### 3.1. The Baseline Characteristics

A total of 72 chronic long-term hemodialysis participants were enrolled in this study and categorized into two groups based on their serum IAA levels: the low IAA group (IAA < 0.5 μg/mL) consisting of 35 participants with an average age of 57.6 years, and the high IAA group (IAA ≥ 0.5 μg/mL) comprising 37 participants with an average age of 60.9 years. No significant differences were observed between the two groups in age, sex, hemodialysis vintage, clinical biochemistry data, or proton pump inhibitor use (Table 1). The distribution of IAA levels is shown in Supplementary Figure S1.

### 3.2. The Gut Microbiota Diversity Index Was Associated with IAA Levels

**Figure 1:**
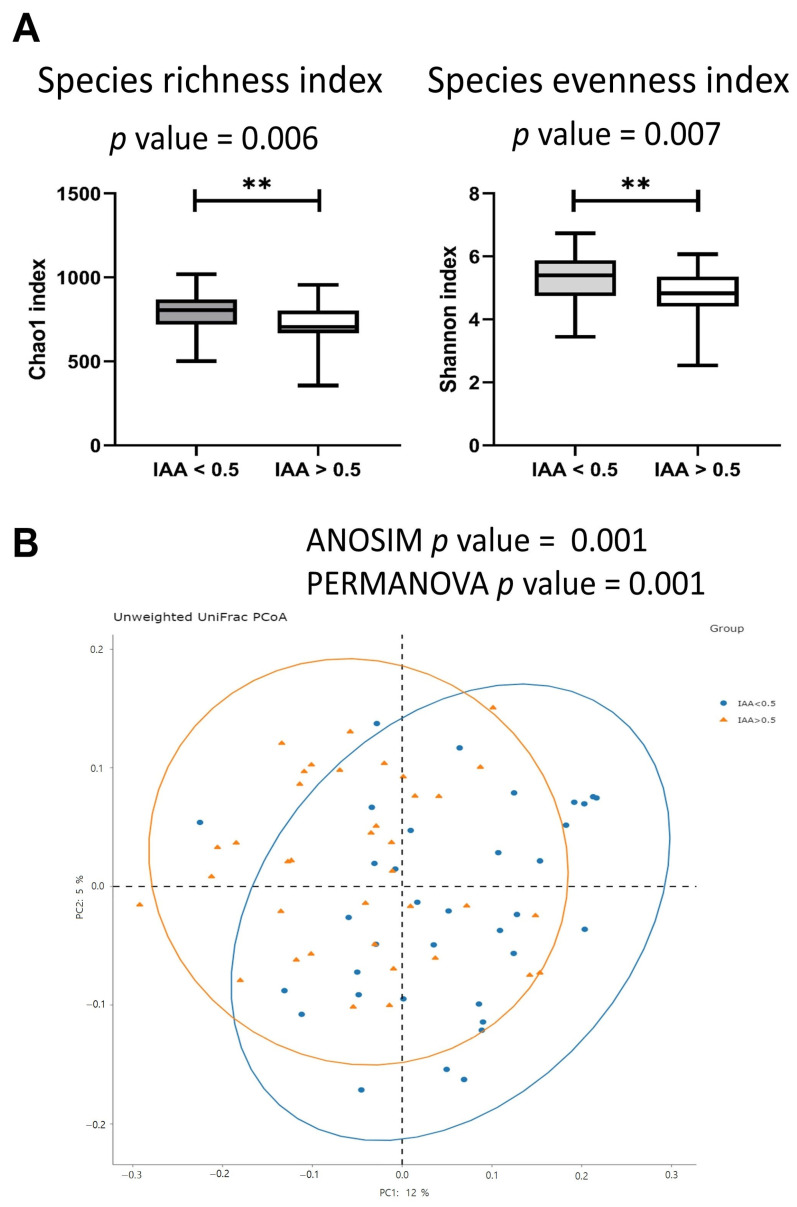
The α-diversity and β-diversity in hemodialysis patients with high and low levels of indole-3-acetic acid (IAA). (**A**) Patients with high IAA levels presented a higher α-diversity (species richness index and species evenness index) than patients with low IAA levels. The star mark ** referred to *p*-value <0.05 (**B**) Patients with high IAA levels had a different β-diversity (Bray–Curtis index) compared to patients with low IAA levels. The β-diversity *p*-value was calculated using homogeneity of group dispersions by analysis of similarities (ANOSIM) test and permutational multivariate analysis of variance (PERMANOVA) test.

Patients exhibiting high IAA levels demonstrated lower α-diversity in their gut microbiome, as evidenced by lower values of both the Chao 1 diversity index and the Shannon diversity index, compared to patients with low IAA levels (Figure 1A). Moreover, in terms of β-diversity, patients with high IAA levels exhibited a significant distinction in the Bray–Curtis index compared to those with low IAA levels, as determined by the similarity-based analysis of similarities (ANOSIM) test and permutational multivariate analysis of variance (PERMANOVA) test (Figure 1B).

### 3.3. The Microbial Features Associated with IAA Levels

**Figure 2:**
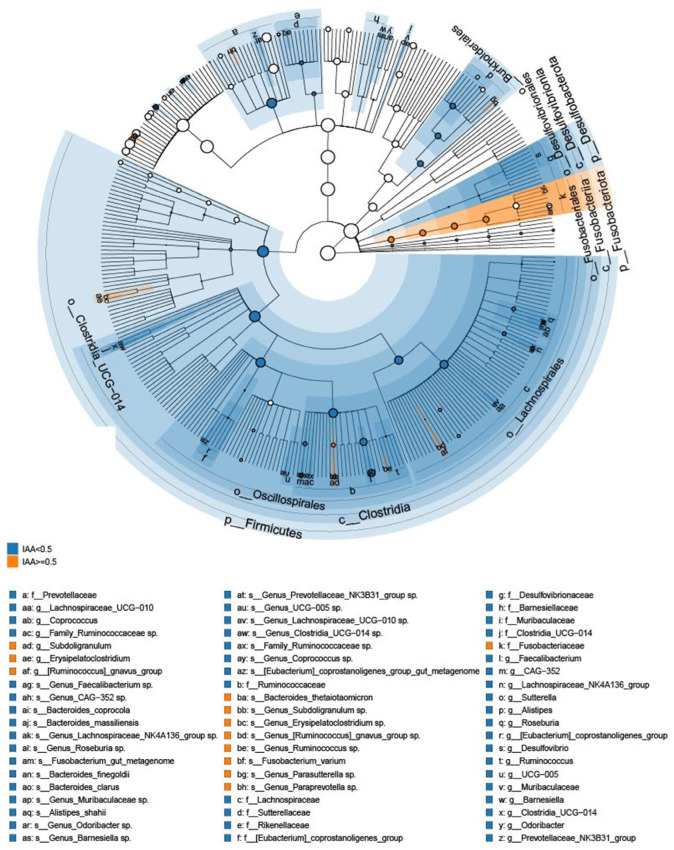
Taxonomic differences were detected between patients with low and high levels of indole-3-acetic acid. Cladogram showing differentially abundant taxonomic clades with an LDA score > 3.0 among patients with low and high levels of indole-3-acetic acid.

**Figure 3:**
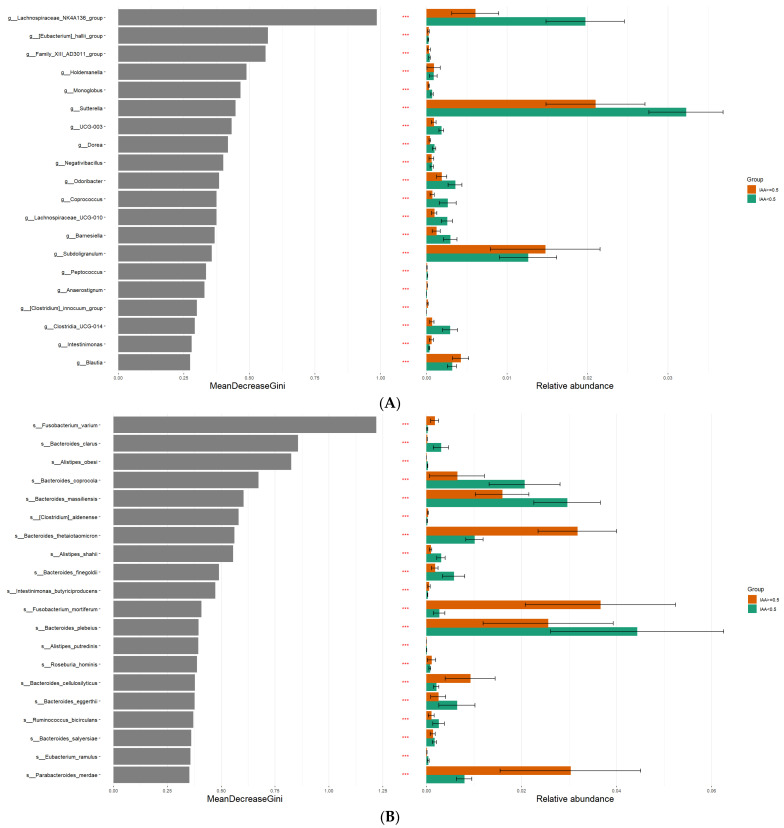
Determination of bacteria specific for discrimination across low and high levels of indole-3-acetic acid in hemodialysis patients by applying random forest analysis at the (**A**) genus-level abundance and (**B**) species-level abundance. The star sign *** referred to the significant difference in abundance with *p*-value < 0.05.

**Figure 4:**
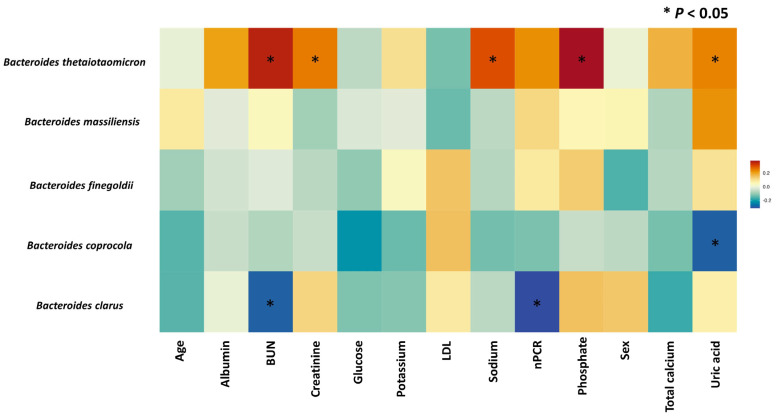
The correlation between IAA-associated *Bacteroides* species and sex, age, albumin, BUN, creatinine, glucose, potassium, LDL, sodium, nPCR, phosphate, total calcium, and uric acid in hemodialysis patients. * indicated *p* < 0.05.

The gut microbiome features associated with patients exhibiting low and high IAA levels were analyzed by LefSe analysis to investigate bacterial taxa significantly enriched in the high- or low-IAA groups. The cladogram illustrating the tree associations between taxonomy and IAA levels is shown in Figure 2, and the detailed microbial taxa are depicted in Supplementary Figure S2. The high-IAA group was enriched in the following taxa: family Fusobacteriaceae; genera *Subdoligranulum*, *Erysipelatoclostridium*, *Ruminococcus*, and *Parasutterella*; and species *Fusobacterium varium* and *Bacteroides thetaiotaomicron*. Random forest cross-validation analyses at the genus (Figure 3A) and species (Figure 3B) levels revealed the top 20 taxa predicting IAA levels. Among these, the *Bacteroides* genus emerged as the most associated with IAA levels. Notably, within the *Bacteroides* genus, four species were enriched in individuals with low IAA levels: *Bacteroides clarus*, *Bacteroides coprocola*, *Bacteroides massiliensis*, and *Bacteroides finegoldii*. Conversely, one species, *B. thetaiotaomicron*, was enriched in individuals with higher IAA levels (Figure 2 and Figure 3). Furthermore, the heatmap demonstrated the correlation between these five species and sex, age, albumin, BUN, creatinine, glucose, potassium, LDL, sodium, nPCR, phosphate, total calcium, and uric acid (Figure 4). Notably, *B. thetaiotaomicron* showed a significant positive correlation with BUN, creatinine, sodium, phosphate, and uric acid, while *B. clarus* exhibited a significant negative association with BUN and nPCR. Additionally, *B. coprocola* demonstrated a negative correlation with uric acid (Figure 4).

## 4. Discussion

Within the cohort of 72 patients undergoing long-term hemodialysis, those in the higher-IAA group (IAA ≥ 0.5 μg/mL) displayed reduced α-diversity in the gut microbiome, as indicated by lower values in the Chao 1 diversity index and the Shannon diversity index. A statistically significant difference in β-diversity was also noted between the two patient groups with different IAA levels. The combination of LEfSe and random forest analysis revealed that the genus *Bacteroides* was most associated with IAA levels. Four of the species analyzed were enriched in individuals with low IAA levels, including *B. clarus*, *B. coprocola*, *B. massiliensis*, and *B. finegoldii*, whereas *B. thetaiotaomicron* was enriched in individuals with higher IAA levels. These findings offer valuable insights into the potential roles of the gut microbiota in modulating IAA levels and contribute to a deeper understanding of the intricate interplay between gut microbiota and IAA metabolism.

IAA is a tryptophan metabolite processed by the gut microbiota and is a protein-bound uremic toxin that cannot be adequately eliminated in patients undergoing hemodialysis. Consequently, this leads to the accumulation of toxins and functional alterations in the gut microbiota, contributing to the development of chronic systemic diseases. IAA is a specific ligand of AhR that can activate the AhR signaling pathway [23], thereby helping to regulate intestinal immunity and inflammation and maintain intestinal homeostasis [24]. A previous study reported that IAA is potentially involved in regulating cell proliferation, hematopoietic cells, and the Janus kinase (JAK)–signal transducer and activator of transcription (STAT) signaling pathway [5]. Understanding the intricate interplay between IAA and the gut microbiota is paramount to gaining insights into potential therapeutic targets for regulating IAA levels in patients with kidney disease. To the best of our knowledge, this study represents the first attempt to investigate and evaluate the link between gut microbiota composition, especially several specific *Bacteroides* species, and IAA levels in hemodialysis patients, offering novel perspectives for comprehending the role of the gut microbiota in IAA metabolism.

In previous studies, alterations in the gut microbiome have been observed in individuals undergoing hemodialysis [6], with hemodialysis patients often exhibiting decreased diversity of the gut microbiota [6]. Furthermore, in the present study, a higher level of IAA was associated with reduced microbiota diversity, potentially attributed to gut dysbiosis. This condition directly relates to increased intestinal permeability due to the deterioration of the epithelial barrier, leading to chronic systemic inflammation [25]. Furthermore, gut dysbiosis can result in elevated production of uremic toxins, such as trimethylamine (TMA), and other harmful metabolites while decreasing beneficial metabolites like short-chain fatty acids (SCFAs) [26]. Consequently, these metabolic disturbances caused by gut dysbiosis may contribute to the progression of kidney disease. Hence, the relationship between gut dysbiosis and kidney disease is bidirectional, as kidney disease can lead to changes in gut microecology.

Numerous studies have investigated the association between specific species and kidney disease but few have focused on the species associated with IAA levels in kidney disease. Tryptophan conversion into indole is primarily controlled by the enzyme tryptophanase [27], which has been detected in approximately 27 genera associated with the gut [28]. Indeed, several gut bacteria, including *Clostridia*, *Bacteroides*, and *Escherichia* produce IAA through tryptophan metabolism [29]. The present study revealed that *Bacteroides* was most associated with IAA levels, with four species (*B. clarus*, *B. coprocola*, *B. massiliensi*, and *B. finegoldii*) associated with a lower level of IAA and one species (*B. thetaiotaomicron*) with a higher level of IAA. *B. thetaiotaomicron* is a human symbiont that stabilizes the colon ecosystem [30] and synthesizes large amounts of spermidine and putrescine in the cecum of pectin-fed gnotobiotic rats [31]. Pectin is a soluble indigestible polysaccharide that stimulates cecal polyamine formation in rats [31]. It has been shown that the cecal contents of mono-associated rats fed a fiber-free diet contained large amounts of spermidine, the major polyamine [31]. IAA is the precursor of indole-3-aldehyde and 3-methylindole, and 3-methylindole is formed by the decarboxylation of IAA and produced by *B. thetaiotaomicron* [32]. Furthermore, a tryptophanase gene has been identified in *B. thetaiotaomicron* which is absent in other *Bacteroides* species. The gene BT1492 is prevalent among the gut metagenomes of healthy humans and correlated with *Bacteroides* abundance. *B. thetaiotaomicron*, unlike other *Bacteroides* species, can produce indole when cultured in tryptophan-rich conditions and a *B. thetaiotaomicron* mutant lacking the BT1492 gene showed no indole production, affirming its role in *Bacteroides* indole production [33]. Therefore, our investigation yielded a noteworthy revelation, as we observed a positive association between *B. thetaiotaomicron* and IAA levels but also a negative correlation between circulating IAA levels and four specific microbial species, namely *B. clarus*, *B. coprocola*, *B. massiliensis*, and *B. finegoldii*. This intriguing observation gains additional support from the Gusty atlas database [34], highlighting *B. clarus* as being negatively associated with blood IAA levels in healthy subjects. Our findings suggest that individual *Bacteroides* species may exert distinct effects on IAA levels at the species or sub-species level but further research is warranted to explore the underlying mechanisms and implications of these interactions.

The findings demonstrating associations between specific *Bacteroides* species and IAA levels suggest that the microbiome composition may impact circulating levels of this uremic toxin, which may in turn influence clinical outcomes. For example, *B. thetaiotaomicron* was positively correlated with uremic toxins, creatinine, sodium, phosphate, and uric acid, while other *Bacteroides* species showed negative correlations with some markers. These correlations hint that *B. thetaiotaomicron* enrichment and lower levels of other *Bacteroides* species may promote increased production and reduced clearance of uremic toxins and metabolites linked to worse cardiovascular outcomes. In contrast, higher abundances of *B. clarus*, *B. coprocola*, and *B. massiliensis* appear protective, correlating with reduced uremic toxin burden. Further mechanistic studies in animal models or clinical interventions modulating the gut microbiota are needed to determine if promoting the growth of beneficial *Bacteroides* species and limiting *B. thetaiotaomicron* could improve IAA metabolism, reduce systemic uremic toxin load, and translate to better clinical outcomes for CKD and dialysis patients. Ultimately, targeted manipulation of the gut microbiota and IAA pathways may offer therapeutic promise.

The strength of this study lies in its focused examination of the gut microbiome in hemodialysis patients and its impact on IAA levels. It used multiple algorithms to identify associated features; employing both LefSe and random forest approaches provides greater confidence in determining influential taxa. Thus, exploring correlations between individual bacteroid species and clinical variables can further support the clinical relevance of the microbiome findings, in particular the key species like *B. thetaiotaomicron*. Nevertheless, some limitations inherent in this study should be acknowledged. Firstly, the sample size was relatively small, concentrating solely on the hemodialysis population. Nonetheless, it is noteworthy that our study stands as the inaugural exploration into the interplay between IAA and the gut microbiota within hemodialysis patients. Amplifying the cohort with a larger sample size would undoubtedly offer a more comprehensive portrayal of microbiome alterations. Secondly, the utilization of a cross-sectional approach inherently restricts the establishment of causality. Nevertheless, we successfully identified robust associations between IAA levels and gut microbiota composition. To substantiate these findings and comprehend the migratory dynamics of the microbiome, future endeavors must encompass longitudinal investigations and follow-up assessments, encompassing both CKD and hemodialysis populations. Thirdly, we postulated an inverse relationship between IAA levels and clinical prognosis with a direct influence of shifts within the gut microbiota. It is essential to acknowledge that our analysis was conducted without continuous sampling of fecal specimens, limiting our capacity to discern the dynamic fluctuations therein. Notably, the dietary guidelines for CKD (pre-dialysis) patients diverge from those of dialysis patients, transitioning from low-protein to high-protein regimens as the condition progresses. This dietary evolution contributes to a uremic milieu within the gut lumen, triggering intricate changes that are still inadequately comprehended. Fourthly, the absence of a control group (such as patients with kidney disease not undergoing hemodialysis or healthy individuals) could limit the ability to draw definitive conclusions about the specific impacts of hemodialysis on gut microbiota and IAA levels. Lastly, a follow-up study is needed to enhance our understanding of these interlocking modifications. It is crucial to periodically assess fecal samples, capturing the microbial composition at various stages and facilitating a comprehensive, long-term assessment.

## 5. Conclusions

Our study demonstrated that a higher level of IAA was linked to reduced gut microbiota diversity, with five *Bacteroides* species being associated with circulating IAA levels.

## Figures and Tables

**Table 1:** Baseline characteristics of hemodialysis participants with high and low levels of indole-3-acetic acid.

	IAA < 0.5 μg/mL (*n* = 35)	IAA ≥ 0.5 μg/mL (*n* = 37)	*p*-Value
Age, Mean (SD)	57.6 (10.8)	60.9 (9.7)	0.171
Male, *n* (%)	20.0 (57.1%)	20.0 (54.1%)	0.792
Hemodialysis vintage, months, Mean (SD)	83.0 (66.8)	95.2 (83.0)	0.497
Proton pump inhibitor used, *n* (%)	5.0 (14.3%)	5.0 (13.5%)	0.925
Clinical biochemistry, Mean (SD)			
Albumin (g/dL)	4.0 (0.4)	3.9 (0.3)	0.482
LDL cholesterol (mg/dL)	107.7 (30.1)	101.4 (26.3)	0.343
Fasting glucose (mg/dL)	113.4 (38.0)	105.8 (32.5)	0.361
Blood urea nitrogen (mg/dL)	63.9 (11.5)	65.8 (14.2)	0.539
Creatinine (mg/dL)	10.9 (2.1)	10.7 (1.8)	0.655
Uric acid (mg/dL)	7.1 (1.1)	6.8 (1.7)	0.386
Sodium (mmol/L)	137.4 (2.3)	137.2 (2.6)	0.703
Potassium (mmol/L)	4.6 (0.7)	4.4 (0.6)	0.180
Total calcium (mg/dL)	9.2 (1.0)	9.3 (0.9)	0.574
Phosphate (mg/dL)	4.8 (0.9)	4.6 (1.1)	0.480
nPCR (g/kg/day)	1.1 (0.2)	1.2 (0.2)	0.079

LDL, low-density lipoprotein cholesterol; nPCR, normalized protein catabolic rate.

## Data Availability

The data will be provided upon request to the corresponding author.

## Supplementary Materials

The following supporting information can be downloaded at: https://www.mdpi.com/article/10.3390/biomedicines12010148/s1, Figure S1: The distribution of indole-3-acetic acid (IAA) levels. Figure S2: Linear discriminative analysis (LDA) effect size (LEfSe) analysis between patients with low (blue) and high (orange) levels of indole-3-acetic acid.
