# Supplementary material for: Exploring the Relationship between Gut Microbiome Composition and Blood Indole-3-acetic Acid in Hemodialysis Patients
Source: Biomedicines. 2024 Jan 10;12(1):148. doi: 10.3390/biomedicines12010148 (PMC10813781; doi:10.3390/biomedicines12010148)

Supplemental Materials for

“Exploring the relationship between gut microbiome composition and blood indole-3-acetic acid in hemodialysis patients”

Supplementary Figure 1. The distribution of Indole-3-acetic acid (IAA) levels.

Supplementary Figure 2. Linear discriminative analysis (LDA) effect size (LEfSe) analysis between patients with low (blue) and high (orange) levels of indole-3 acetic acid.

Supplementary Figure 1. The distribution of Indole-3-acetic acid(IAA) levels.

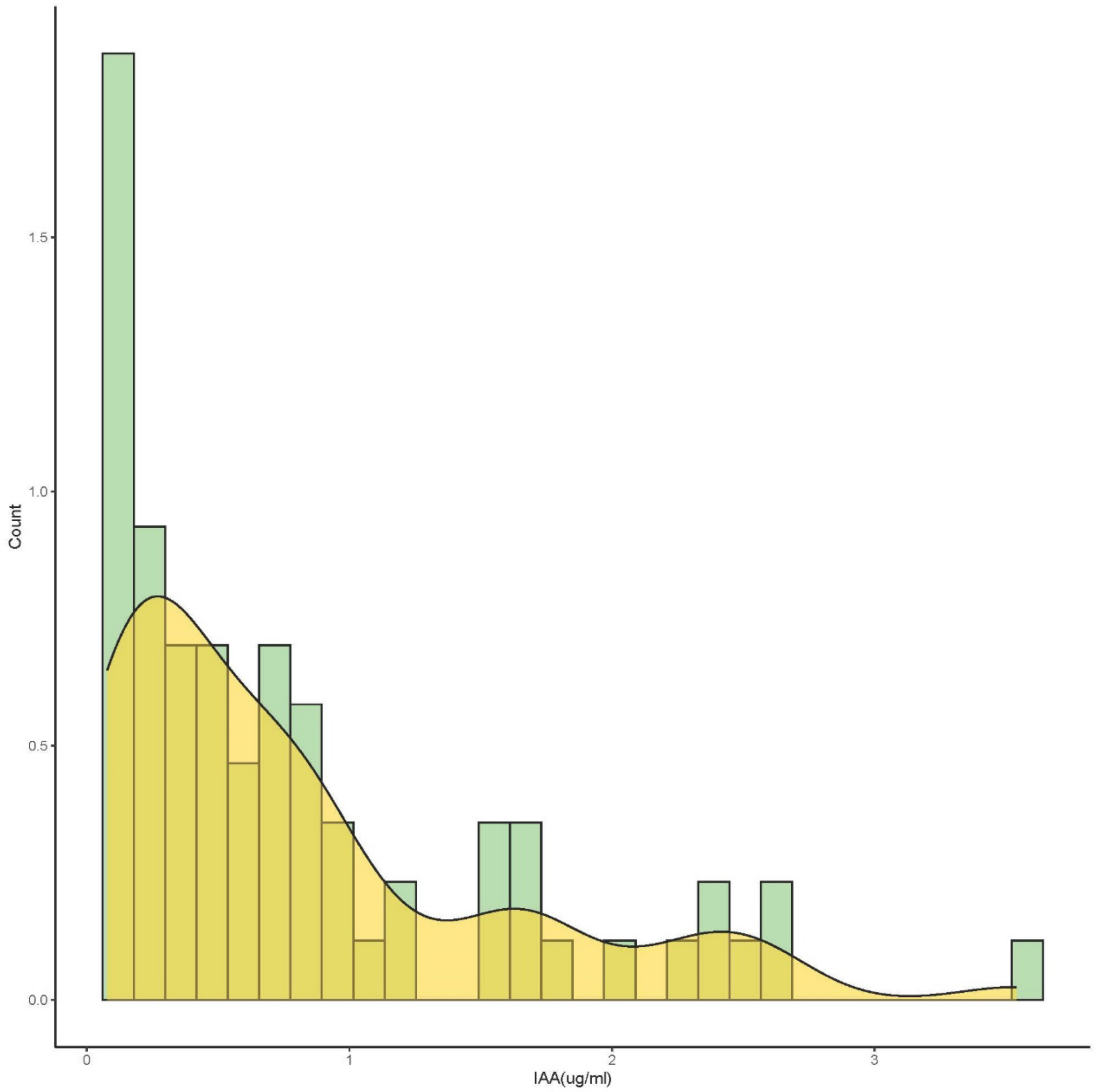

Supplementary Figure 2. Linear discriminative analysis (LDA) effect size (LEfSe) analysis between patients with low (blue) and high (orange) levels of indole-3 acetic acid.

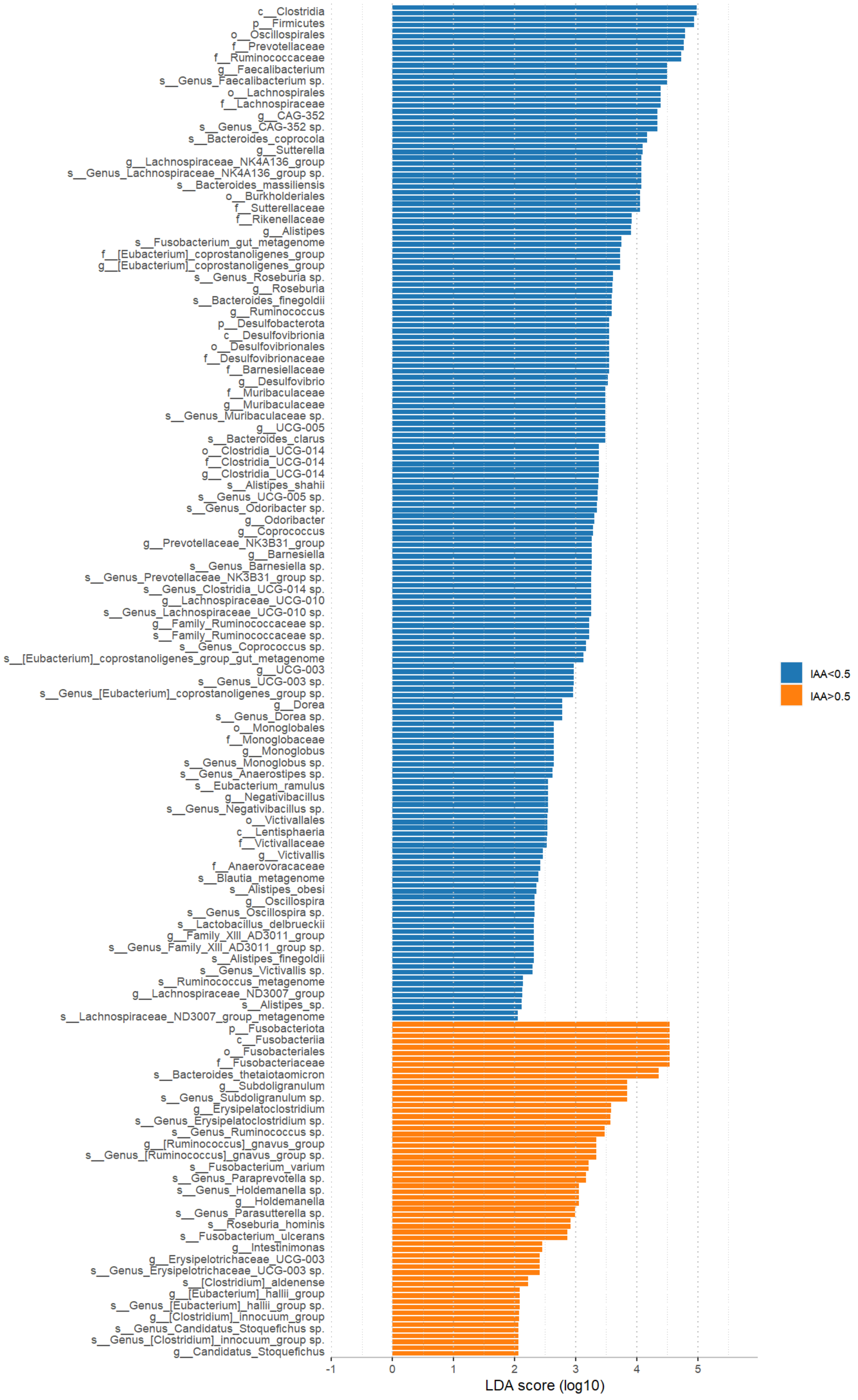

Supplement: Supplementary file 1 [file biomedicines-12-00148-s001.zip › biomedicines-2783607-supplementary.pdf]
